# Cytomegalovirus‐associated esophagitis on early esophageal cancer in immunocompetent host: a case report

**DOI:** 10.1186/s13099-021-00418-4

**Published:** 2021-04-16

**Authors:** Daisuke Murakami, Hideaki Harada, Masayuki Yamato, Yuji Amano

**Affiliations:** 1 Department of Gastroenterology, New Tokyo Hospital, 1271 Wanagaya, Matsudo, Chiba, 270-2232 Japan; 2 Institute of Advanced Biomedical Engineering and Science, Tokyo Women’s Medical University, 8-1, Kawada-cho, Shinjuku-ku, Tokyo, 162-8666 Japan; 3 Department of Gastroenterology, Tokyo Women’s Medical University Yachiyo Medical Center, 477-96 Owadashinden, Yachiyo, Chiba, 276-8524 Japan; 4 Department of Endoscopy, New Tokyo Hospital, 1271 Wanagaya, Matsudo, Chiba, 270-2232 Japan

**Keywords:** Cytomegalovirus, Early esophageal cancer, Endoscopic submucosal dissection, Programmed death‐ligand 1

## Abstract

**Background:**

Cytomegalovirus (CMV)-associated gastrointestinal diseases usually occur in immunocompromised patients; however, few cases has also been described in healthy hosts despite still unclear pathological mechanisms. CMV esophagitis causes various lesions, such as erythematous mucosa, erosions, and ulcers, although such inflammatory changes can appear in superficial esophageal cancers or in surrounding areas. CMV-associated esophagitis has been also reported in cancer patients, but typically in those with advanced and/or terminal stage cancers secondary to chemoradiotherapy-induced immunosuppression or the physiologic demands of the malignancy itself. To our best knowledge, we firstly report on an immunocompetent patient subject to endoscopic submucosal dissection (ESD) for early esophageal cancer complicated with CMV infection.

**Case presentation:**

A 77-year-old man underwent esophagogastroduodenoscopy (EGD) at a local clinic. EGD revealed a lugol-unstained reddish lesion with whitish exudates in the middle-distal esophagus. Histological evaluation of lesion biopsy revealed atypical squamous epithelium with CMV-positive granulation tissue and aggregates of macrophages, prompting referral for further examination and treatment. Magnifying endoscopy with narrow-band imaging showed an erosive lesion with white moss in a well-demarcated brownish area with irregular mesh-like microvessels. ESD was performed for diagnosis and treatment. Histopathological examination of the resected specimen revealed superficial, moderately differentiated squamous cell carcinoma (SCC) with multiple lymphatic infiltration, and few CMV-positive cells were found in the erosive part of the SCC. Interestingly, he had no underlying conditions to predispose to CMV infection and no risk factors for esophageal cancer, other than gender and age. He received neither steroids for stricture prevention nor antiviral agents post-EGD and 4-month follow-up was negative for esophagitis.

**Conclusions:**

This is the first report of a case of CMV esophagitis superimposed on early esophageal cancer in an immunocompetent host and might provide valuable information for possible adverse effects of steroid administration during ESD procedures, despite their common use for prevention of post-ESD stricture.

## Background

Human cytomegalovirus (CMV) infections in children and young adults are associated with virus latency, with opportunistic infections in adulthood typically associated with immunosuppressed states [1]. In such immunocompromised hosts, CMV infection often evokes pneumonia, gastroenteritis, retinitis and hepatitis with organ-specific symptoms [1, 2]. Various CMV-associated gastrointestinal (GI) diseases manifesting as erythematous mucosa, erosions, and ulcers have been endoscopically observed in the GI tract from the mouth to the anus [3]. CMV-associated GI tract lesions, including esophagitis, usually occur in immunocompromised patients, such as those with acquired immunodeficiency syndrome and those receiving organ transplantation or corticosteroid therapy [3, 4], although CMV esophagitis in healthy hosts has been reported despite still unclear pathological mechanisms [5, 6]. CMV-associated esophagitis is also reported in cancer patients, but typically in those with advanced and/or terminal stage cancers secondary to chemoradiotherapy-induced immunosuppression or the physiologic demands of the malignancy itself [7–9].

Here, we present the first reported case of CMV esophagitis on superficial esophageal cancer in an immunocompetent individual. This case also highlights the importance of considering the adverse effects of steroids after extensive endoscopic treatment of esophageal cancer in cases involving CMV or other infections [10]. Moreover, we suggest this clinical case is not only rare but also implicative for understanding the unknown mechanism between cancer immunity, representative of programmed death-ligand 1 (PD-L1)/PD-1 molecule [11], and CMV infection.

## Case presentation

**Fig. 1:**
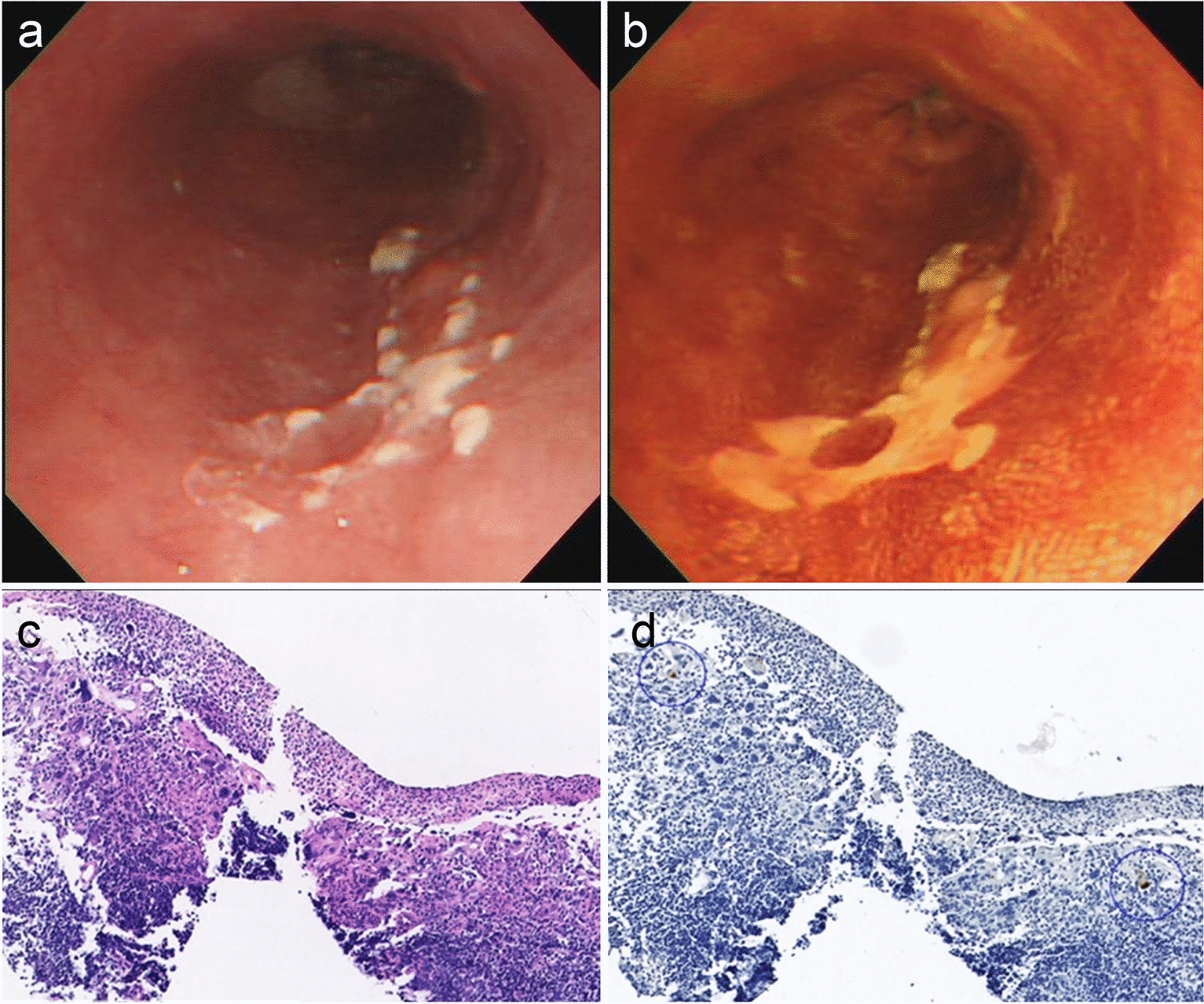
EGD and the biopsy results at a local clinic. **a** EGD with conventional white-light imaging revealed a 20-mm flat, reddish lesion with multiple white spots on the posterior wall of the middle-distal esophagus. **b** Lugol chromoendoscopy revealed a well-demarcated unstained lesion. **c** Histology of the biopsy specimen revealed atypical squamous epithelium and granulation tissue with intranuclear inclusion bodies. **d** Immunohistochemical staining revealed intranuclear inclusions positively stained with anti-CMV antibodies (blue circles)

**Fig. 2:**
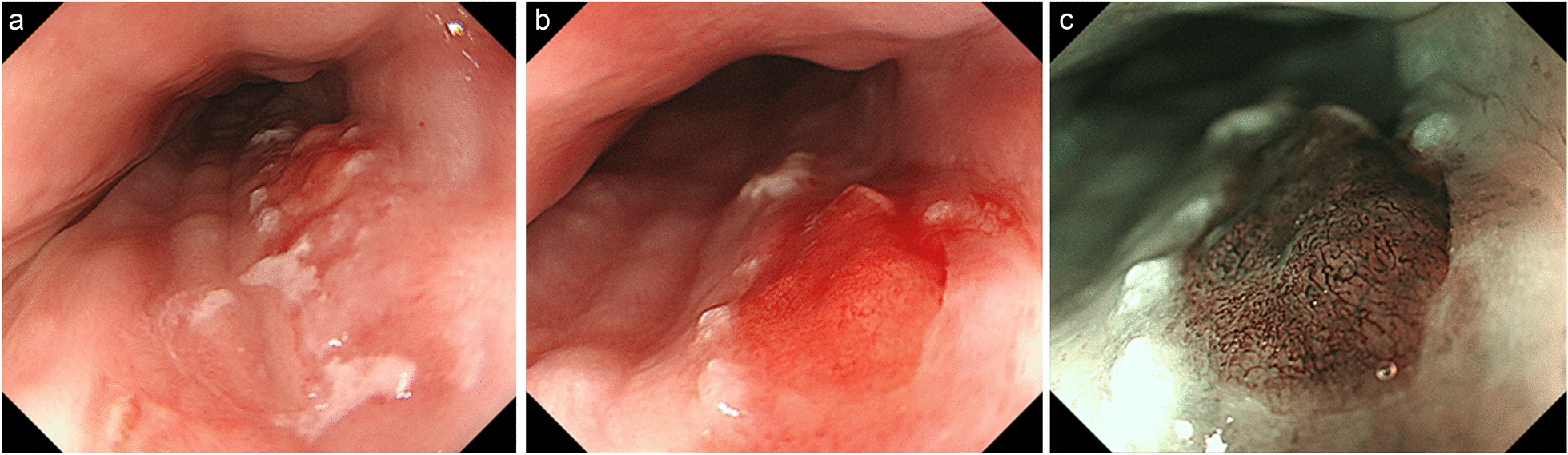
Repeated EGD in our hospital using magnifying endoscopy with NBI. **a** A decrease in the adherence of white spots compared with the previous EGD (Fig. 1a). **b** Magnifying endoscopy revealed a network of fine capillaries on the erosive part of the lesion. **c** Using magnifying endoscopy with NBI, the erosive lesion was recognized as a well-demarcated brownish area with intervascular background coloration and the microvessels revealed irregular mesh-like pattern, namely type B2i

**Fig. 3:**
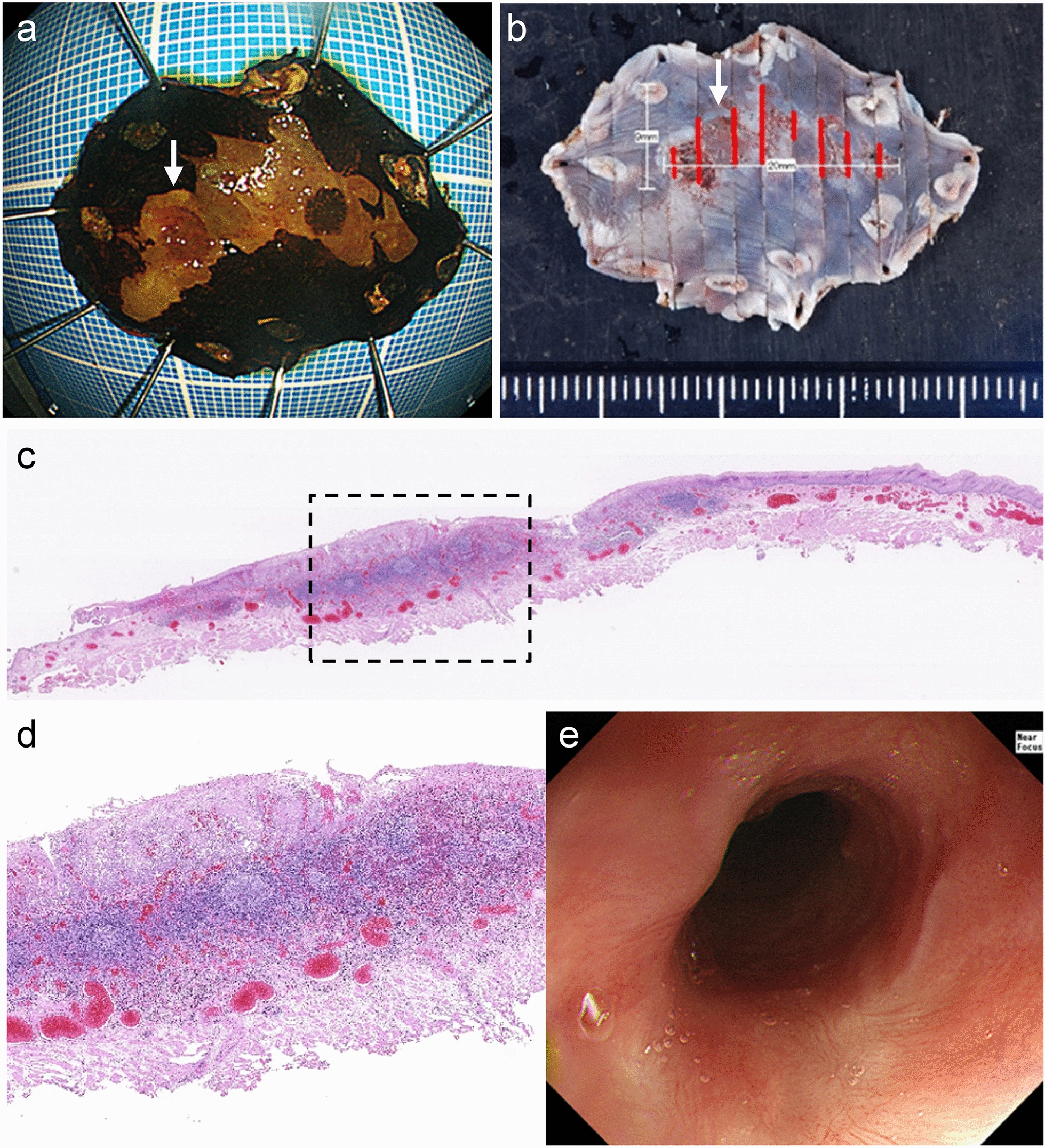
The analysis of the lesion performed by ESD and the follow-up EGD. **a** Grossly, the resected specimen with erosion (arrow) measured 38 × 26 mm and the Lugol-voiding lesion was 20 × 9 mm in size. **b** The resected specimen was divided into fourteen parts. Squamous cell carcinoma defined by histology was almost consistent with the Lugol-voiding area (red line). The arrow was accordant with a macroscopic erosion. **c** Histopathological examination revealed moderately differentiated squamous cell carcinoma confined to EP and LPM, and both the lateral and vertical resection margins were tumor-free (hematoxylin–eosin stain, Loupe view). **d** Medium-power view of the area outlined by the black square in part **c**. Hyperplastic lymphoid follicles were observed in the lowest part of the tumor. **e** At the 4-month follow-up EGD, the scar revealed no traces of esophagitis or ulceration. (Conventional white-light image)

 A 77-year-old man underwent esophagogastroduodenoscopy (EGD) at a local clinic. EGD with conventional white-light imaging revealed a slightly depressed reddish lesion with whitish exudate in the middle-distal esophagus (Fig. 1a). Lugol chromoendoscopy showed an iodine-unstained lesion (Fig. 1b). A biopsy of the reddish lesion revealed atypical squamous epithelium with CMV-positive granulation tissue and aggregates of macrophages (Fig. 1c, d), prompting referral to our department for further evaluation and treatment. He was a social drinker and never-smoker, and otherwise had no underlying conditions to predispose to CMV infection and no risk factors for esophageal cancer, other than gender and age. Serologic evaluation was notable for positive anti-CMV IgG, negative anti-CMV IgM, and negative CMV antigens (C7-HRP). Lymphocyte count and CD4/CD8 ratios were normal, and the serological assay of HIV was negative. EGD at our institute revealed a well-demarcated erosive lesion at 31–33 cm from the upper incisors (Fig. 2). The adherence of white spots had decreased compared with that observed in previous EGD (Fig. 2a). Magnifying endoscopy with narrow-band imaging (NBI) showed a vascular pattern suggestive of inflammatory, namely type B2i, vessels concerning for malignancy per the Japan Esophageal Society (Fig. 2b, c) [12]. Chromoendoscopy revealed an iodine-unstained area on the oral side of the lesion, but pink-color change of the lesion itself, prompting diagnostic treatment by endoscopic submucosal dissection (ESD). En bloc resection of the lesion was successfully performed (Fig. 3a, b). Histopathological examination revealed a moderately differentiated squamous cell carcinoma (SCC) with invasion of the lamina propria mucosae (LPM) without lymphatic metastasis, and both lateral and vertical resection margins were tumor-free (Fig. 3c). Hyperplastic lymphoid follicles forming germinal centers were observed in the deeper part of LPM and submucosa (Fig. 3d). Immunohistochemical staining of CMV was also performed in the resected specimen, although CMV-infected cells were not identified by the pathologist at that time. In this case, steroid and antiviral agents (i.e. ganciclovir) were not administrated. At the 4-month follow-up EGD, the resected scar did not reveal traces of esophagitis or esophageal ulceration, and there were no obvious signs of CMV infection (Fig. 3e).

**Fig. 4:**
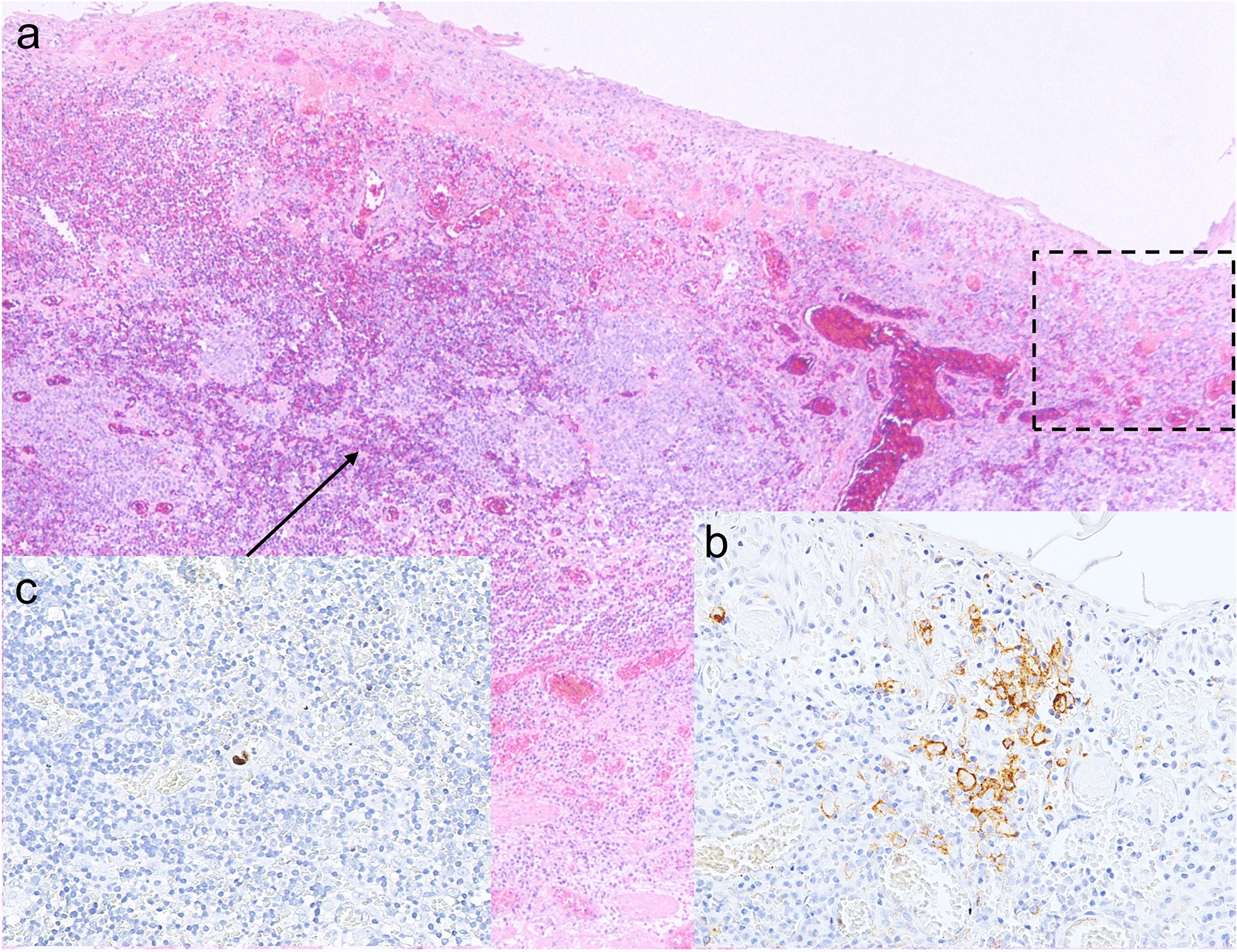
Histological and immunohistochemical analysis of the ESD specimen. **a** HE staining in the erosive part of the SCC into fourteen-pieces (Fig. 3b arrow). **b** Magnified view of the PD-L1 expressing area outlined by the black square in **a**. A small amount of PD-L1 positive cancer cells was partially observed in only the limited region. **c** Only a few CMV-positive cells were found in the vicinity (corresponding area, arrow) of the PD-L1 expressing cancer cells at the serial section of **b**

In the current case, the presence of endoscopic erosion with white moss and pathological CMV-positive cells led to the diagnosis of CMV esophagitis on early esophageal cancer, despite no evidence of systematic immunosuppressive disease. The most interesting point is whether there is any relationship between CMV esophagitis and early cancer, or whether this association was found simply by chance. Based on prior data pertaining to the relationship between CMV infection and anti-tumor immunity, we hypothesized that PD-L1/PD-1 signaling, well-known immunoinhibitory molecules of cancer immunity, might also be involved in our patient. Therefore, we performed immunohistochemical staining with anti-PD-L1 antibody (clone SP142) of the ESD specimen. Only partial staining of PD-L1 (less than 1% of the whole lesion) was observed within a limited region of the specimen (Fig. 4a, b). Interestingly, the PD-L1 expressing cancer cells were consistent with the erosive part of the cancer (see Fig. 3c, arrow). When focused on the PD-L1 positive cells of the tissue sections, a few CMV-infected cells were found in the vicinity of the PD-L1 expressing cancer cells at the serial section (Fig. 4b), prompting the pathologist to amend the final diagnosis to include CMV infection on the ESD specimen. On re-examination, the remainder of the specimen was negative for CMV-infected cells. Ultimately, based on our pathological diagnosis, we were able to raise suspicion about the correlation between PD-L1-related tumor immunity and CMV infection.

## Discussion and conclusions

CMV esophagitis generally exhibits various types of endoscopic appearances (e.g., erosion, erythema, ulceration), although mucosal ulceration is the most commonly reported finding [13]. The standard approach for diagnosis is endoscopic biopsy of the inflammatory findings for histological confirmation of intranuclear inclusions of pathology and positive CMV immunostaining. In our current case, we assumed the encountered erosion with white moss might be secondary to underlying CMV esophagitis, although such inflammatory changes can appear in early SCC or in surrounding areas. These inflammatory findings within SCC are widely known to endoscopists, and some experts separately classify the irregular vessels of SCC with inflammation into type B2i as a subtype of typical B2-cancerous vessels [12]. Importantly, the erosion and white spots on SCC in this case are endoscopically indistinguishable from the characteristics of CMV infection or common pattern SCC with inflammation. There is currently no data pertaining to CMV detection in esophageal cancer tissue, although the possibility of CMV infection had been long recognized in various tumor tissues [14]. Interestingly though, there is increasing evidence suggesting that CMV is associated with colorectal cancers with one study revealing positive CMV PCR detection in 42.3 % (69/163) of colorectal cancer cases [15, 16]. Since the most common location of CMV-associated GI tract lesions is the colon followed by the esophagus and stomach [1, 3], it is not unreasonable that CMV infection might hide in the esophageal cancer tissue. Our case, however, highlights the fact that CMV may be detected in early esophageal cancers, rather than limited to only advanced cases and should warrant further investigation. Moreover, this case highlights the importance for the endoscopic community to consider possible adverse effects of administering steroids to post-ESD patients with esophageal SCC and inflammatory findings. Since esophageal stricture caused by extensive ESD requires frequent treatment with painful balloon dilatation, prophylactic use of steroids has been widespread in clinical practice for preventing the stricture [10, 17]. Simple oral intake and/or a local injection of steroids are clinically the most practical and useful way for controlling post-ESD stricture development [17] despite several alternative methods including cultured oral mucosa epithelial cell sheet transplantation [18], biodegradable stents [19], and endoscopic radial incision and cutting [20]. Since this case lesion was dissected at half-circumference, we did not use steroids after ESD. We speculate that CMV esophagitis was cured along with ESD, as no other medical treatment including antiviral agents was required and post-ESD follow-up EGD was negative for esophagitis. However, had steroids been administered, we would have run the risk of CMV reactivation of submucosal CMV-infected cells. CMV esophagitis is also known to occur with severe submucosal inflammation, increasing the risk for perforation and stenotic scarring [21, 22]. CMV-positive cells are typically observed as fibroblasts, endothelial cells and immune cells in granulation tissue rather than squamous epithelium [23]. Therefore, CMV-infected cells might remain in the submucosa after ESD and progress to CMV-associated GI diseases if oral steroid or local steroid injection to ulcer bed were used.

Our case also brings up an importance notion in that CMV esophagitis is rarely observed in immunocompetent hosts, as our patient above [1, 3, 5–7]. CMV esophagitis in advanced cancer patients is often attributed to the chemoradiotherapy-induced immunosuppression or the physiologic demands of the malignancy itself [24]. In early cancers, however, the cancer cannot be considered as a cause of immunosuppression. In our case, the patient had neither systematic symptoms nor systematic immunosuppressive disease. The serological results suggested he previously infected CMV. Anti-CMV IgM is generally detected in primary infection, though can be sometimes elevated in reactivation of the latent CMV infection. CMV antigenemia is also known as useful for the diagnosis of active CMV infection, however, it was reported that the sensitivity of CMV antigens in CMV GI diseases of immunocompromised patients was 65.4% [25]. Serology is not sufficient to make a timely diagnosis of CMV infection, and the absence of CMV-IgM antibody and antigenemia may not exclude CMV infection in an immunocompetent individual. Therefore, we assumed local reactivation of the latent CMV infection in this case, although the possibility of local re-infection cannot be denied. Based on these findings, we hypothesize that our patient’s CMV esophagitis developed due to a relationship with cancer immunity, particularly the reported correlation between CMV infection and PD-1 blockage therapy for certain cancers. Recently, a clinical case report presented a patient with metastatic bladder cancer resistant to treatment with PD-1/PD-L1 inhibitors, later found to have active CMV gastritis. Interestingly, after recovery from CMV infection, she achieved complete remission by resumed treatment of PD-1 inhibitor, despite progressive disease prior to developing CMV gastritis [26]. In addition, in experimental CMV-infected tumors in a mouse model, not only anti-tumor but anti-viral T cells infiltrated the tumor and expressed PD-1 [27]. Infecting CMV into the growing tumor combined with anti-PD-L1 therapy led to synergized clearance of more than 60% of the tumor [28]. These data suggest that immune cells induced by CMV infection might reflect the immune status of the tumor micro-environment and contribute to clearance of cancer cells [29]. Considering that the efficacy of PD-1 inhibitors was also demonstrated in patients with esophageal SCC, but survival benefit was not correlated with patients’ level of tumor PD-L1 expression [30], CMV reactivation might be a key factor of cancer immunity via PD-L1/PD-1 signaling. In our data, a very small amount of CMV-positive cells was incidentally observed near the PD-L1 expressing cancer cells, though they had been missed by primary pathologic examination. This clinical course is of great interest, though whether the PD-L1 expression of SCC is linked with CMV reactivation or not remains unclear.

In summary, this is the first case report of CMV esophagitis encountered upon superficial esophageal cancer in an immunocompetent host. This case might provide valuable messages for endoscopists when considering whether to administer steroids or not in patients post-ESD, although only this single case should not make referred to the management of ESD of esophageal lesions. Furthermore, we hope that this case will lead to discussions regarding the correlation between CMV infection and cancer immunity.

## Data Availability

All data generated or analysed during this study are included in this published article.

## Abbreviations

CMV: Cytomegalovirus
GI: Gastrointestinal
PD-L1: Programmed death-ligand 1
EGD: Eesophagogastroduodenoscopy
ESD: Endoscopic submucosal dissection
SCC: Squamous cell carcinoma
LPM: Lamina propria mucosa
HIV: Human immunodeficiency virus
NBI: Narrow-band imaging
